# The Robust Vessel Segmentation and Centerline Extraction: One-Stage Deep Learning Approach

**DOI:** 10.3390/jimaging11070209

**Published:** 2025-06-26

**Authors:** Rostislav Epifanov, Yana Fedotova, Savely Dyachuk, Alexandr Gostev, Andrei Karpenko, Rustam Mullyadzhanov

**Affiliations:** 1 Department of Mathematics and Mechanics, Novosibirsk State University, Novosibirsk 630090, Russia; i.antonevich@g.nsu.ru (Y.F.); sawel.nsk@gmail.com (S.D.); rustammul@gmail.com (R.M.); 2 Meshalkin National Medical Research Center, Novosibirsk 630055, Russia; dr.gostev@gmail.com; 3 Scientific Research Institute of Physical-Chemical Medicine, Moscow 119435, Russia; andreikarpenko@rambler.ru; 4 Institute of Thermophysics, Novosibirsk 630090, Russia

**Keywords:** vessel centerline extraction, one-stage centerline reconstruction, vessel segmentation, multitask neural network, computed tomography angiography images, vascular modeling toolkit

## Abstract

The accurate segmentation of blood vessels and centerline extraction are critical in vascular imaging applications, ranging from preoperative planning to hemodynamic modeling. This study introduces a novel one-stage method for simultaneous vessel segmentation and centerline extraction using a multitask neural network. We designed a hybrid architecture that integrates convolutional and graph layers, along with a task-specific loss function, to effectively capture the topological relationships between segmentation and centerline extraction, leveraging their complementary features. The proposed end-to-end framework directly predicts the centerline as a polyline with real-valued coordinates, thereby eliminating the need for post-processing steps commonly required by previous methods that infer centerlines either implicitly or without ensuring point connectivity. We evaluated our approach on a combined dataset of 142 computed tomography angiography images of the thoracic and abdominal regions from LIDC-IDRI and AMOS datasets. The results demonstrate that our method achieves superior centerline extraction performance (Surface Dice with threshold of 3 mm: 97.65%±2.07%) compared to state-of-the-art techniques, and attains the highest subvoxel resolution (Surface Dice with threshold of 1 mm: 72.52%±8.96%). In addition, we conducted a robustness analysis to evaluate the model stability under small rigid and deformable transformations of the input data, and benchmarked its robustness against the widely used VMTK toolkit.

## 1. Introduction

Medical visualization techniques are instrumental in the diagnosis and management of vascular pathologies, including aortic aneurysms, femoral artery occlusions, and coronary artery disease. Morphological information obtained from vascular medical images provide critical support for enabling preoperative planning [1,2], image-guided surgical interventions [3,4], and postoperative assessment [4]. For instance, the precise measurements of aortic diameters and lengths at anchoring zones are pivotal for selecting optimal endograft dimensions and configurations. Notably, inadequate preoperative sizing directly correlates with an elevated risk of type 1A endoleak due to compromised device-wall apposition [5]. Similarly, quantification of maximal aortic diameters, bifurcation angles, and vessel tortuosity serves as a cornerstone for clinical decision-making [6]. In image-guided aortic interventional surgery, centerline extraction is required for preoperative path planning, particularly in cases involving complex aortic anatomy. Furthermore, accurate reconstruction of vascular geometry is essential for patient-specific hemodynamic modeling. Two primary types of mathematical models are utilized: (1) three-dimensional (3D) fluid dynamics models [7], which depend on a precise 3D vascular reconstruction derived from segmentation, and (2) one-dimensional (1D) fluid flow models [8], which necessitate both segmentation and extraction of a centerline graph representing vascular tubular networks.

Computed tomography angiography (CTA) is the most widely adopted procedure for visualizing vessels in human organs in vivo. Segmentation and centerline extraction are essential steps in image-based vascular modeling and morphological analysis. In recent years, convolutional neural networks have been widely utilized in medical image processing due to their minimal manual intervention and high accuracy [9]. Li et al. [10] developed a 2D cascaded convolutional network for contour extraction on cross-sectional 2D images, followed by 3D reconstruction of the aortic wall and lumen using pre-extracted centerlines. For the segmentation of large vessels, Dou et al. [11] proposed a 3D supervised deep learning model capable of segmenting volumetric structures, including the heart, aorta, and liver. Cao et al. [12] segmented the entire aorta using a 3D U-Net and subsequently differentiated the true and false lumens via an additional U-Net. Epifanov et al. [13] introduced a single 3D U-Net architecture with a ResNeXt encoder for simultaneous segmentation of the lumen, an aortic wall with thrombotic masses, and calcifications, highlighting that calcifications constitute the most challenging class for segmentation. Tailored augmentation techniques implemented by authors help to mitigate the complexity of their detection. Cepero et al. [14] introduced SeqSeg (sequential segmentation), a deep learning-based algorithm for automatic tracing and segmentation of vascular structures from medical images, which effectively generalizes to unseen vascular structures not included in the training data. Although the aforementioned methods can accurately segment the vessel lumen with reduced reliance on empirical parameters, they do not directly provide centerlines. Vessel tracking is an effective strategy for extracting structural information.

Centerline provides a concise representation of the vessel topology. An ideal centerline extraction algorithm produces points that are closely aligned with the geometric centers of the object cross-sections, accurately capture all true branches of the structure, and avoid generating spurious or false-positive branches. Traditional centerline extraction methods can be broadly categorized into three main classes [15]: minimal cost path, thinning, and tracking methods.

Voronoi diagram-based methods extract centerlines by identifying paths within the Voronoi diagram that minimize the integral of the radii of maximal inscribed spheres along the trajectory. This approach has been specifically implemented in the Vascular Modeling Toolkit (VMTK) [16], a reference package for vascular segmentation and automatic centerline extraction [17,18,19,20]. Distance mapping methods [21] are commonly used to construct the shortest path between two points. The process typically begins with computing a distance-from-source map, which encodes the distance from a specified source point to each voxel within the 3D object. The shortest path to the source is then obtained by following the gradient descent of this map from the target point. However, this approach does not guarantee that the path remains centered within the object. A similar strategy can be applied to compute skeletons using distance-from-boundary (DFB) fields [22]. To improve centeredness, a penalty term can be added to the cost function at each node to steer paths away from the object boundaries. This issue can be effectively addressed by using the DFB field as node weights and constructing a minimum-cost spanning tree build from the DFB field. Nevertheless, methods of this class rely on manually defined features, heuristic rules, and explicit specification of branch endpoints, thereby limiting their degree of automation and generalizability [21].

Topological thinning methods iteratively remove voxels located on the boundary of the shape while preserving both connectivity and overall topology [23]. To enforce the centeredness of the resulting skeleton, voxel removal is typically guided by the voxel distance to the boundary, with those farther from the center removed first. However, centerlines extracted by these methods often come along with spurious branches and usually need post-pruning [24].

Tracking-based techniques extract the medial axis by iteratively estimating and predicting the next optimal voxel to be included in the centerline path. A variety of tracking schemes have been proposed, including image intensity-based methods such as ridge tracking [25], inertia matrix-guided tracking [26], and local maxima approaches [27]. Additionally, model-based techniques were suggested to estimate the medial line, including the use of B-spline curves [28] and super-ellipsoids [29]. As an alternative, Sironi et al. [30] reformulated centerline extraction as a regression problem, while Schneider et al. [31] proposed multivariate Hough regression forests to simultaneously extract binary segmentations and centerlines. In traditional approaches, post-processing is typically employed following centerline extraction to remove spurious branches, reconnect discontinuous segments, and smooth or interpolate the resulting trajectories [32,33]. In summary, despite the wide range of preprocessing, segmentation, and skeletonization techniques proposed in the literature for vascular structures, no universally optimal pipeline exists.

Recent studies have proposed several algorithms based on deep learning methods. Tetteh et al. [34] introduced a neural network with an original convolutional architecture designed to perform vessel segmentation, centerline extraction, and bifurcation detection through three subsequent binary classification tasks. A conceptually similar approach is discussed in Kromm et al. [35], who also proposed a network for retinal vessel segmentation and centerline generation based on a Capsule Network inspired by the Inception architecture [36]. However, these methods usually do not take into account the direct constraint relationship between the two tasks.

The multitask learning (MTL) paradigm has garnered considerable attention in recent years [37]. Rather than developing separate models for individual tasks, MTL unifies them within a single architecture, thereby enhancing performance through knowledge transfer across tasks [38,39,40,41,42,43]. Most studies in this field have focused on coronary and retinal arteries [44,45,46]. Shit et al. [47] introduced a centerline Dice (clDice) loss function to realize the constraint of the centerline on blood vessels, but they only focused on the segmentation task, and the centerline is only an intermediate result. In contrast, Pan et al. [48] proposed MSC-Net, a multitask learning framework designed for simultaneous retinal vessel segmentation and centerline extraction, leveraging clDice loss for both tasks. Guo et al. [49] proposed a two-head multitask fully convolutional network (FCN) which simultaneously generates a locally normalized distance map and a list of branch endpoints for coronary arteries. Rouge et al. [50] presented a multitask cascaded network with a U-Net backbone for cerebrovascular segmentation, where the subsequent skeletonization task directly benefits from the segmentation output and may also leverage information from the input image. The application of MTL to diverse vascular structures demonstrates enhancement of performance in joined tasks, underscoring the methodological promise of the approach for this study.

This paper proposes a multitask network to combine the two tasks, blood vessel segmentation and centerline extraction. We specially designed the hybrid network with convolutional and graph layers and loss function to better discover the topological relationship between these two tasks and realize their complementary features. The network enables the end-to-end approach capable of directly predicting connected centerline points with real-valued coordinates, thereby eliminating the post-processing steps required by algorithms that generated centerlines either implicitly presented or without connectivity points. We evaluated the accuracy of our method against existing open-source solutions, including classical and neural network-based approaches, and demonstrated its superior performance. Additionally, we conducted a robustness analysis to assess the algorithm stability under small affine and elastic perturbations of the input image, and benchmarked its robustness against that of the widely used VMTK tool.

## 2. Materials and Methods

### 2.1. General Pipeline

**Figure 1:**
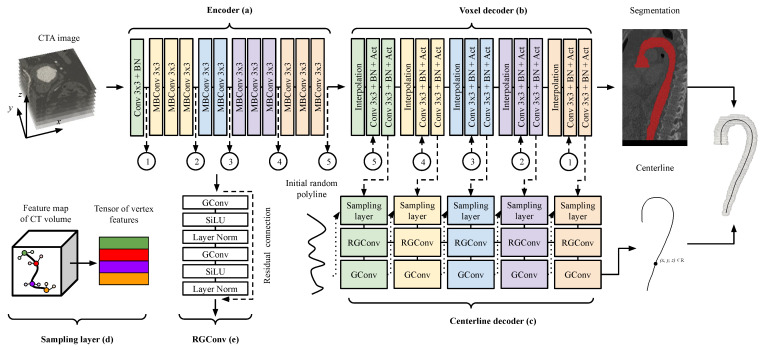
Overview of the proposed hybrid network.

We have developed a multitask neural network that simultaneously addresses the interrelated tasks of segmentation and centerline extraction of blood vessels from input CTA images. The schematic diagram is presented in Figure 1. The network architecture is hybrid, incorporating convolutional layers to extract feature maps from CTA images and graph-based layers to transform the extracted features into centerline coordinates. Structurally, the architecture can be divided into three modules: an encoder (Figure 1a), a voxel decoder (Figure 1b), and a centerline decoder (Figure 1c). Each of these modules are split into five stages to make cross connections between modules. The neural network accepts an input CTA image of dimensions H×W×D, which is processed through the encoder to generate latent features of the CTA image with reduced dimension H’×W’×D’. To generate a vessel segmentation mask, the latent features are passed to a voxel decoder, which produces an output array of dimensions H×W×D. Each voxel in this array corresponds to the predicted probability of belonging to the vessel region. Simultaneously, these latent features are also input to the centerline decoder. Within the centerline decoder, sampling layers extract features corresponding to centerline points, using coordinates derived from the preceding stage. Subsequently, graph layers transform these features into three-dimensional coordinates of centerline points. In the first sampling layer, the three-dimensional polyline is randomly initialized in real coordinates. Thus, the proposed neural network enables simultaneous segmentation of lumen and reconstruction of the vessel centerline through a multitask architecture.

### 2.2. Neural Network Architecture

In selecting the architecture for the voxel encoder, we focused on convolutional architectures. On the one hand, they require less training data to achieve comparable performance than transformer-based models, which is particularly important given the difficulty of obtaining high-quality medical annotations. On the other hand, convolutional architectures are also less demanding in terms of computational resources, which is especially relevant for three-dimensional data. We chose the EfficientNetV2 architecture [51] as the backbone for our encoder because this architecture was selected through an automated architecture search process, optimally balancing performance quality and memory consumption. However, in Appendix A, we also provide performance measurements for other widely used convolutional architectures, such as ResNet [52] and DenseNet [53]. We adopted a layer composition similar to that used in the b0-sized model, as this variant is most suitable for processing data at a resolution of 224 pixels, which closely matches the dimensions of the used data in this study. Additionally, the encoder is divided into five blocks to facilitate skip connections to the decoders. A detailed schematic of the encoder is illustrated in Figure 1a. Mobile inverted bottleneck convolution (MBConv) following the original choice consists of convolution layer, squeeze and extraction block and convolution layer. The voxel decoder was constructed utilizing the same layers as those implemented in our prior study [13]. Each block incorporates an interpolation layer, followed by a twice-repeated sequence of a convolutional layer, a batch normalization layer, and a ReLU activation layer. After the interpolation layer, the upsampled feature map is concatenated with the feature map derived from the voxel encoder. This composition layer mirrors the U-Net architecture, which is widely recognized for its performance in segmentation tasks. We employ a voxel encoder–decoder framework to achieve segmentation of the vessel lumen.

The neural network-based methods for segmentation and centerline extraction discussed earlier rely on voxel-wise target predictions, such as label maps [34] and distance maps [54]. These target formats inherently limit the ability to reconstruct centerlines at subvoxel resolution without the aid of additional post-processing steps, including filtering, smoothing, and point interpolation. However, the use of such post-processing techniques to achieve subpixel accuracy introduces an additional source of error in the localization of centerline points, which is further compounded by potential inaccuracies in the neural network output. To address these limitations, we propose a graph-based architecture that circumvents voxel-level constraints by directly predicting centerline points with continuous real-valued coordinates. During model initialization, a randomly generated polyline serves as the starting structure, enabling the extraction of initial centerline point features via a sampling layer. Appendix B provides an analysis of the network performance depending on the initial polyline initialization. Based on the coordinates of candidate centerline points ($pts^{c}$), the sampling layer retrieves their corresponding feature representations ($pts^{f}$) from the latent features (*x*) of the CTA image produced by the voxel decoder. To incorporate contextual information surrounding each point, a convolutional layer predicts the neighbors ($nbrs^{c}$) of the candidate points, and a unified representation of each point and its local neighborhood ($nbhd^{f}$) is subsequently constructed as follows:

(1) $$\begin{array}{cc} \; & pts^{f}=\mathrm{grid}\_\mathrm{sample}\left(x,pts^{c}\right),\\ \; & nbrs^{c}=\mathrm{Conv}\left(pts^{f}\right),\\ \; & nbrs^{f}=\mathrm{grid}\_\mathrm{sample}\left(x,nbrs^{c}\right),\\ \; & nbhd^{f}=\mathrm{Conv}\left(\left[pts^{f},nbrs^{f}\right]\right). \end{array}$$

This neighborhood-aware representation is then processed by a RGConv block to refine the topological structure of the centerline. Each RGConv block consists of a main processing path and a residual connection. The architectural design of these blocks draws inspiration from the decoder structure of U-Net [55]. The main path comprises a repeated sequence of a graph convolution layer [56], an activation layer, and a normalization layer called LayerNorm [57]. The SiLU activation function is employed, in line with its use in the encoder architecture. After passing through the RGConv blocks, the final coordinates of the centerline points are predicted using a single graph convolution layer. These coordinates are subsequently utilized in the sampling layers of the following stages to iteratively refine the centerline geometry.

### 2.3. Dataset Preparation

In this study, we employ two publicly available datasets: LIDC-IDRI [58] and AMOS [59]. The LIDC-IDRI dataset comprises 1010 CT volumes, whereas the AMOS dataset contains 500 CT volumes with corresponding segmentation masks of the abdominal aorta. The centerline annotations for these datasets are provided in an external dataset [54], which includes centerline annotations for 101 CT volumes from the LIDC-IDRI dataset and 41 CT volumes from the AMOS dataset. Aortic centerlines were annotated by three independent, experienced radiologists, who placed points in the three-dimensional space by aligning their positions across axial, sagittal, and coronal CT projections [54]. Accordingly, a total of 142 CT volumes (101 from the LIDC-IDRI dataset and 41 from the AMOS dataset) were used in this study, as complete triplets of CT images, segmentation masks, and vessel centerline annotations are publicly available only for this subset. Pixel spacing was uniform across all axes at 2 mm. The x−y plane dimensions ranged from 138 × 138 to 250 × 250 pixels, while the number of *z*-planes varied between 113 and 330. The mean number of centerline points per scan was 31.21 ± 7.88, with an average inter-point distance of 6.03 ± 3.30 mm. The combined dataset of 142 CT images was randomly partitioned into five subsets for cross-validation. To enlarge the effective size of the train subsets in each fold, we augment data with flips, affine and distortion transformations, noising, and blurring [13].

### 2.4. Neural Network Training

The training of our network was supervised by optimizing a compound loss function incorporating ground truth segmentation masks and centerline points. The total training loss was formulated as a combination of voxel and line losses.

#### 2.4.1. Voxel Loss

In the voxel part of the loss, we lean on a combination of focal and dice losses [60,61] as we have done previously during training the network only to make segmentation [13]:

(2) $$\;L_{vox}\left(M^{p},M^{t}\right)=-\sum_{i\in I}\sum_{k\in K}M_{ik}^{t}\left(1-M_{ik}^{p}\right)\log M_{ik}^{p}-\frac{2}{\left|K\right|}\sum_{k\in K}\frac{\sum_{i\in I}M_{ik}^{t}M_{ik}^{p}}{\sum_{i\in I}M_{ik}^{t}+M_{ik}^{p}},$$

where $M_{ik}^{p}$ and $M_{ik}^{t}$ stand for the predicted and ground truth segmentation probabilities for CT volumes with shape *I*. Indexes *i* and *k* iterate over *I* shape and *K* classes, respectively.

#### 2.4.2. Centerline Loss

The centerline loss function is formulated as a composite of three terms. The Chamfer Distance (CD) term minimizes Euclidean distance between predicted and ground truth centerline points:

(3) $$\;L_{cd}\left(C^{p},C^{t}\right)=\sum_{v^{p}\in C^{p}}\min_{v^{t}\in C^{t}}∥v^{p}-v^{t}{∥}_{2}+\sum_{v^{t}\in C^{t}}\min_{v^{p}\in C^{p}}{∥v^{t}-v^{p}∥}_{2},$$

where $C^{p}$ and $C^{t}$ denote the predicted and ground truth centerlines. The iteration over *v* of *C* corresponds to iterating over points of the corresponding centerline. CD is typically used to measure the similarity between two sets of points [62]. A regularization term on the edges encourages a more uniform edge length of the predicted centerline:

(4) $$\;L_{elr}\left(C^{p}\right)=\sum_{e^{p}\in C^{p}}{∥e^{p}∥}_{2},$$

where iteration over $e^{p}$ of $C^{p}$ means iteration over edges of centerline, $e^{p}$ means the average edge length. In the third term of centerline loss, we explicitly leveraged the segmentation head to impose penalties on points situated outside the vessel lumen:

(5) $$\;L_{plr}\left(C^{p},M^{p}\right)=\sum_{v^{p}\in C^{p}}L_{vox}\left({\tilde{M}}_{p}\left(v_{p}\right),1\right),$$

where ˜ means stop-gradient operator, and $M_{p}\left(v_{p}\right)$ denotes sampling with trilinear interpolation from $M_{p}$ by $v_{p}$ coordinates.

#### 2.4.3. Loss Normalization

To normalize the loss between different network heads, we employed exponential averaging of the voxel and centerline losses:

(6) $$\;\bar{L}=\alpha \bar{L}+\left(1-\alpha \right)L,$$

where α is equal to 0.9. We selected the value of 0.9 based on our experiments. The final applied loss for training is equal to:

(7) $$\;L=\bar{L_{vox}}+\bar{L_{cd}+L_{elr}+L_{plr}}.$$

#### 2.4.4. Training Hyperparameters

We trained the model using the AdamW optimizer [63] with a learning rate of $10^{-3}$. The training process comprised 80 epochs, each consisting of 1000 iterations, and was conducted on an NVIDIA (Santa Clara, CA, USA) RTX 3090 Ti GPU with 24 GB of VRAM. The total training time was under six hours, and the inference time per case was less than one minute on a CPU. Prior to input into the neural network, the CTA volumes were windowed and normalized to the range [0, 1]. The windowing process used a level of −200 Hounsfield Units (HU) and a width of 1400 HU. To ensure subpixel accuracy during centerline generation, we fixed the number of points at 96.

## 3. Results

### 3.1. Evaluation Metrics

In this section, we conducted a comparative evaluation of our proposed multitask neural network against both traditional classical methods and existing deep learning-based approaches. In alignment with Yaushev et al. [54], the performance of the centerline generation task was evaluated using one-dimensional adaptations of metrics for comparing sets of points: Surface Dice (SD), Hausdorff Distance (HD), and Average Symmetric Surface Distance (ASSD). Explicit definitions of these metrics are provided in Appendix C. For SD, tolerance thresholds of 1 mm and 3 mm were employed, while HD was calculated at the 95th percentile. We emphasized the SD-3 metric, as it most precisely characterizes the accuracy of centerline reconstruction at the voxel resolution level. This corresponds to the commonly accepted error tolerance of 1–2 voxels, or 2–4 mm, based on the 2 mm voxel spacing of the CT images used. To evaluate subvoxel-level reconstruction precision, we additionally utilized SD-1. The ASSD and HD metrics were applied to quantify the average and maximum distances, respectively, between the extracted and ground truth centerlines. Thus, these metrics enable comprehensive evaluation of both the proportion of accurately reconstructed centerline points at voxel and subvoxel resolutions using SD and the absolute geometric deviations using HD and ASSD, which quantify extreme and average spatial discrepancies, respectively. To evaluate the segmentation task performance quality, we employed the Volumetric Dice (VD), the most widely adopted metric for assessing performance in this task [64].

### 3.2. Comparative Results of Proposed Method with Other Baseline Methods

**Figure 2:**
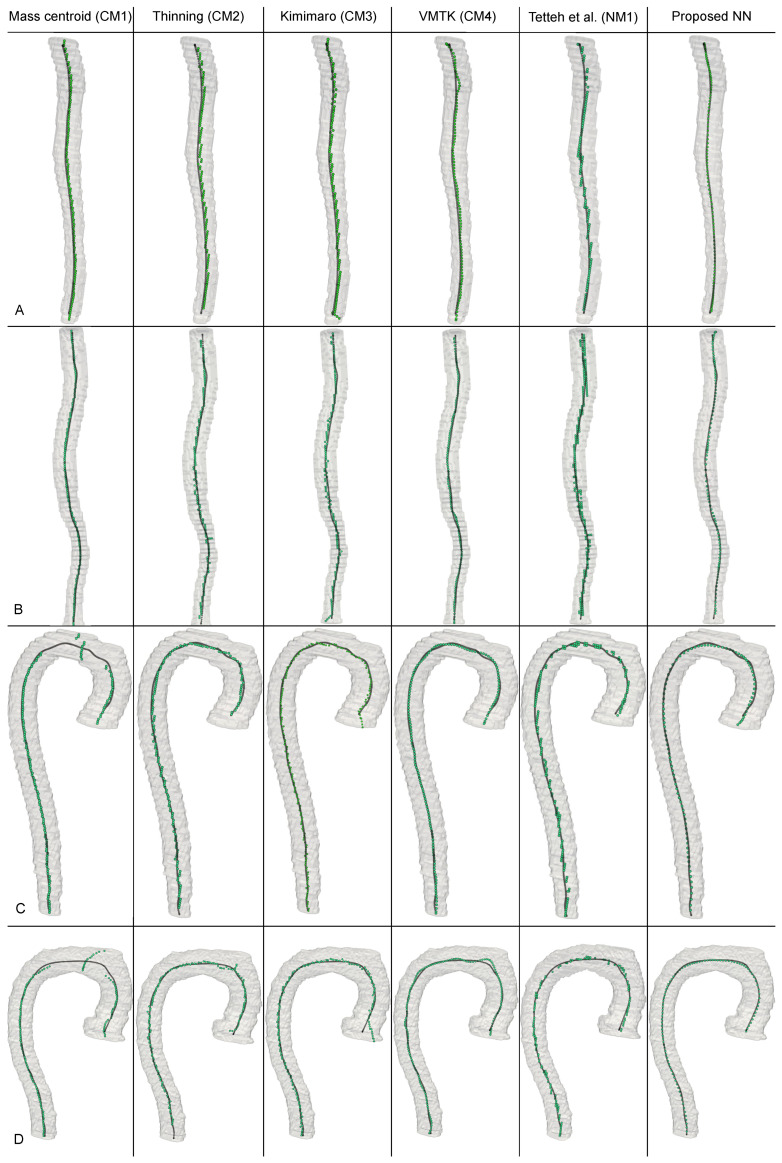
Example centerline reconstructions across different methods [34].

Comparative results are summarized in Table 1. Appendix D provides examples of ground truth segmentations alongside those produced by our algorithm. Figure 2 illustrates representative examples of the algorithms selected for comparison, applied to thoracic and abdominal aortic segments. For benchmarking against classical approaches, we selected the following centerline extraction methods: a mass centroid-based algorithm (CM1) [65], thinning algorithms implemented in scikit-image (CM2) [66], tracking-based algorithms from the Kimimaro library (CM3) [67], and the minimal cost path algorithm from VMTK (CM4) [68]. These algorithms were chosen due to their prevalence in vascular centerline extraction workflows and the accessibility of their reference implementations. In comparative evaluations, our method demonstrated superior performance across all metrics. The mass centroid algorithm achieved acceptable accuracy in extracting centerlines for unbifurcated abdominal aortas (Figure 2 CM1-A, CM1-B). However, it exhibited limitations in the thoracic region due to spurious branch generation during axial slice analysis near the aortic arch (Figure 2 CM1-C, CM1-D). Notably, the mass centroid algorithm yielded an SD-3 score of 89.17%, the lowest among classical evaluated methods. The thinning algorithm exhibited performance comparable to the mass centroid algorithm. While this method did not face fundamental limitations in reconstructing centerlines within the thoracic aorta, achieving an SD-3 score of 92.72%, it remained prone to generating short false branches under certain aortic configurations (Figure 2 CM2-B, CM2-D), which constrained its overall accuracy. Furthermore, as thinning method inherently produces integer-valued points for centerlines, it underperformed in subvoxel precision, yielding an SD-1 score of 58.63% compared to the mass centroid algorithm 64.89%. The Kimimaro algorithm and the Voronoi diagram-based method demonstrated similar performance, achieving SD-3 scores of approximately 95.8%. However, it is critical to note that the VMTK method failed to generate centerlines for 3% of cases (five cases). Thus, its performance metrics were computed only on successfully processed cases. Despite these failures, VMTK offers distinct advantages, such as reconstructing the centerline as a continuous polyline with explicit topological connectivity, a feature absent in other classical methods. The connectivity ensures smooth transitions in the tangent vector to the centerline, whereas Kimimaro generates abrupt directional shifts in the tangential vector, necessitating post-processing procedures for points connecting and trajectory smoothing to enable clinical application in vessel geometric feature computation derived from the centerline. Conversely, while VMTK eliminates the need for post-processing, it requires extensive preprocessing, including surface mesh preparation and explicit endpoint specification, to initiate centerline extraction.

Among neural network-based algorithms, we selected the method proposed by Tetteh et al. [34] (NM1), which employs voxel-wise binary classification, and the approach introduced by Yaushev et al. [54] (NM2), which reconstructs centerlines through the prediction of attraction fields. As we were unable to reproduce the NM2 method, we utilized the quality metrics reported in the original study for comparison [54], which were obtained using the same dataset employed in our research. We attribute this failure to reproduce the method to differences in library versions used in the original studies. The NM1 method demonstrated comparable performance quality to the thinning algorithm while sharing the same fundamental limitations. The NM1 method achieved a quality score of 58.71% on the SD-1 metric and 95.24% on the SD-3 metric. Furthermore, when predicting points, the NM1 method exhibited a tendency to generate point clusters (Figure 2 (NM1-B,NM1-C)), likely attempting to improve the probability of accurate alignment with the true centerline. The NM2 method demonstrated the closest quality metrics to our approach, achieving comparable performance on the SD-3 metric but underperforming on the SD-1 metric. We attribute these outcomes to the fact that the NM2 method, unlike ours, requires complex post-processing steps. To obtain the centerline, the authors emphasize the necessity of employing a non-maximum suppression method to refine the predicted point cloud and using Isomap to establish connections within the refined point cloud. Suboptimal parameter selection for these steps may degrade the final quality of the generated centerline, particularly at subpixel resolution. In contrast, our method eliminates the need for any pre- or post-processing steps. This explains the superior SD-1 metric of 72% achieved by our approach, representing a 16% improvement over NM2. This improvement is further reflected in the ASSD metric, with our method attaining 0.93 mm compared to 1.4 mm for the NM2 method.

To evaluate the impact of MTL on individual task performance, we trained two single-task networks: one specialized exclusively in the segmentation and the other solely in the centerline extraction. In the segmentation task, despite the high performance of the single-task network, the multitask network outperformed its counterpart by 0.26% on the VD metric. In the centerline extraction task, the multitask network demonstrated improvements of 2.51% on the SD-1 metric and 0.94% on the SD-3 metric compared to the single-task network. In our opinion, these enhancements are driven by two key complementary factors. First, the shared layers between the voxel and centerline decoders implicitly provide the latter with access to spatial information about vessel localization. Second, since deviations of generated centerline points outside vessel boundaries are penalized during training, the usage of implicitly known vessel localization is encouraged during centerline generation. The accounting of vessel localization during the generation is further evidenced by a reduction in the HD from 5.85 mm to 2.74 mm. Thus, joint learning ensures knowledge sharing between tasks, thereby enhancing the performance of each individual task.

### 3.3. Robustness Comparison of the Proposed Algorithm and VMTK for Centerline Extraction

We compared the robustness of the proposed neural network against a commonly used Voronoi diagram-based centerline extraction method, implemented in the VMTK library. The method is integrated into medical imaging assessment toolkit, such as 3D Slicer [69] and CRIMSON [18]. To assess robustness, we performed a series of experiments involving data augmentations, such as scaling, rotation, and grid distortion, applied to CTA images and corresponding segmentation masks. During image augmentation, we used B-Spline and Gaussian smoothing interpolation techniques [70,71] for CTA scans and corresponding segmentation masks, respectively. Centerlines were generated using both the neural network and the VMTK method. The neural network successfully constructed the centerlines across all dataset, whereas the VMTK method failed to do so in 7% of cases (ten cases). It should be noted that before applying the image transformations, both methods were able to generate the centerlines successfully.

**Figure 3:**
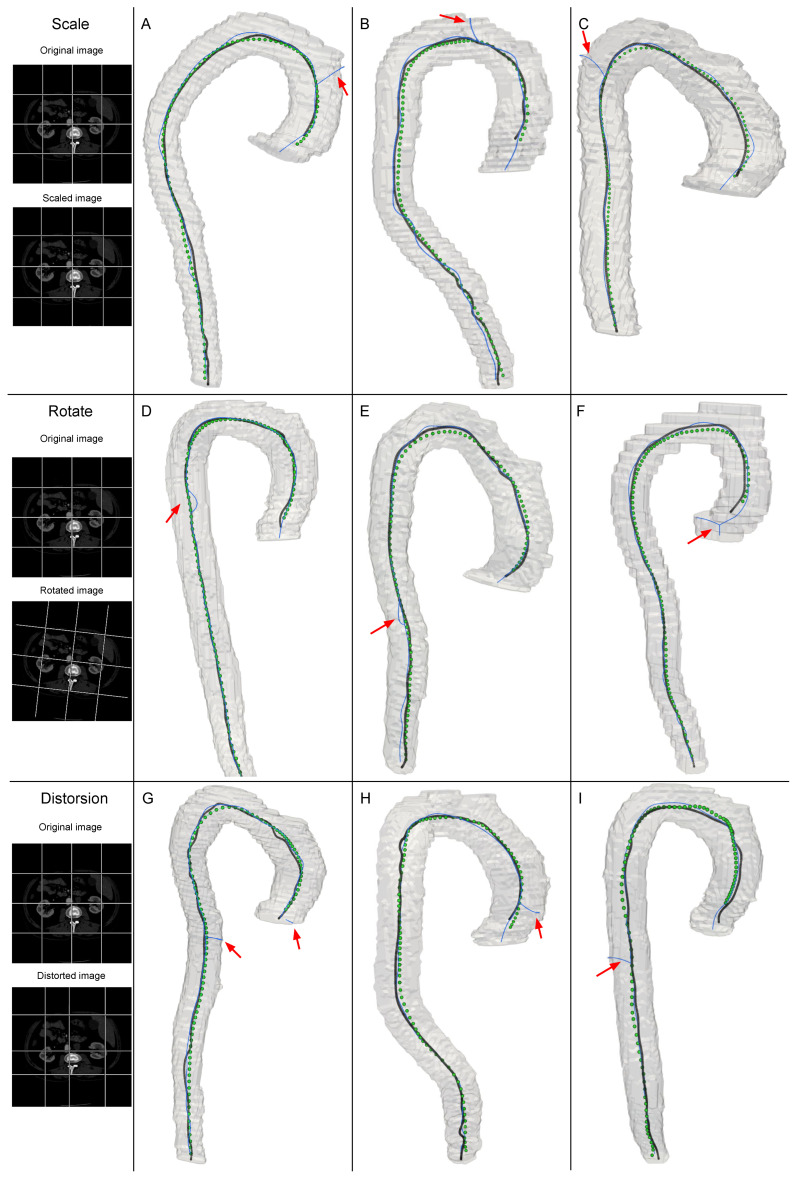
Examples of centerline reconstructions generated by the proposed method and the VMTK for input data subjected to small geometric deformations. White grid on CTA slices used as reference to visualize deformation. Green—proposed method, blue—VMTK, black—ground truth.

The results of centerline extraction for augmented images are presented in Figure 3. Unlike the neural network, which exhibits robustness to input augmentations, the VMTK method demonstrates sensitivity to deformations of segmentation mask induced by augmentation transformations. In 10% of cases produced by VMTK method, erroneous reconstructions were observed, with artifacts categorized as follows: (1) centerline discontinuities resulting in fragmented paths (Figure 3G); (2) topological inaccuracies, such as spurious branches or loops; and (3) significant deviations of the centerline toward the lumen boundary. These failures primarily originated from minor segmentation artifacts, such as spikes, which were reinforced by slight affine transformations of the segmentation masks used to centerline computation. In contrast, the neural network demonstrated robustness to such input image modifications.

### 3.4. Robustness Evaluation of the Proposed Method Under CTA Image Artifacts

**Figure 4:**
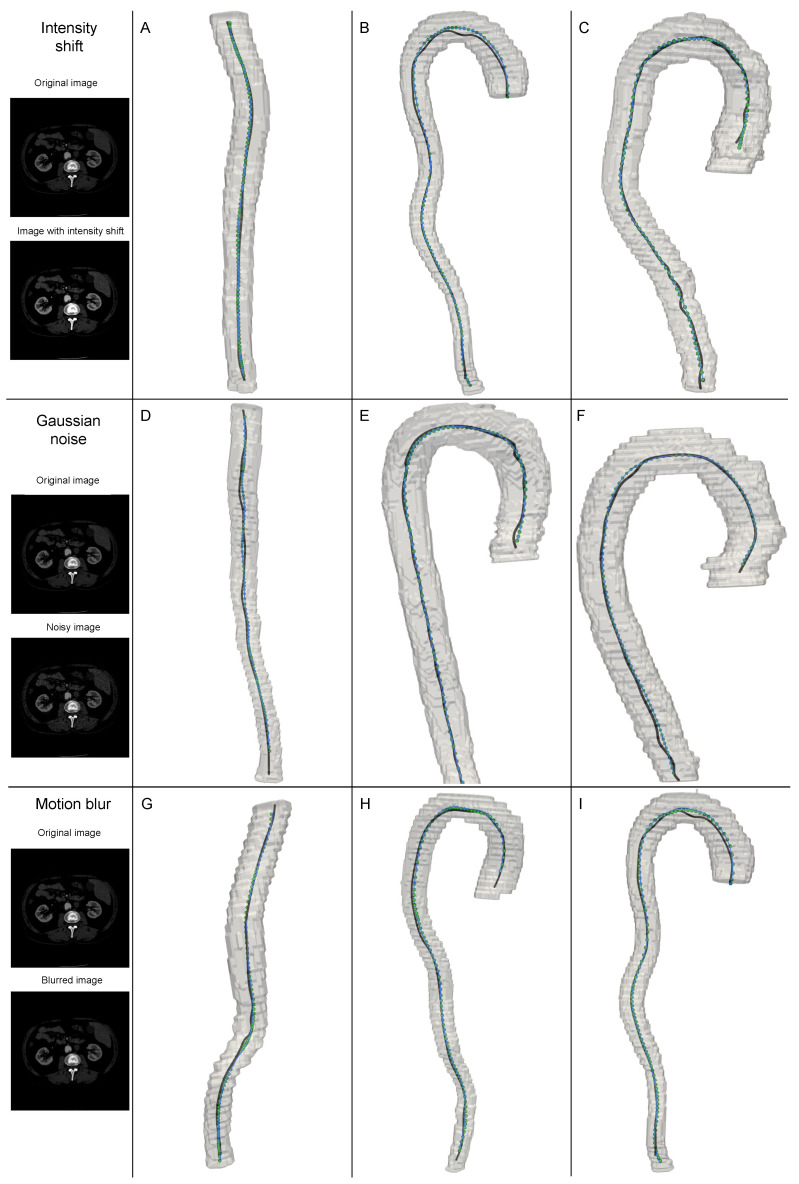
Comparison of centerline reconstructions generated by the proposed method: green—CTA with artifacts, blue—CTA without artifacts, black—ground truth.

In this section, we evaluate the robustness of the proposed method for centerline extraction when applied to CTA images affected by common types of artifacts. We assess model performance under three artifact scenarios: (i) noise-induced artifacts, (ii) calibration-related artifacts, and (iii) motion-induced artifacts caused by patient movement during CT acquisition (Figure 4).

To simulate image noise, additive Gaussian noise with zero mean and a variance of 15 HU was applied. Calibration artifacts were modeled by applying a global linear intensity shift sampled uniformly in the range of [–10, +10] HU, simulating scanner calibration errors. Motion artifacts were emulated using linear motion blur with a displacement amplitude ranging from 1 to 3 voxels.

Quantitative results are summarized in Table 2, which compares the model performance under each artifact type to that on unaugmented (original) data. The model exhibited highly stable performance under noise and calibration artifacts: the SD-3 metric remained above 97.6%, and other metrics, including HD and ASSD, showed negligible variation compared to the baseline. The most pronounced impact was observed in the presence of motion blur, which led to a minor decrease of approximately 1.77% in SD-3 accuracy and slightly increased surface distance metrics. Overall, these results demonstrate that the proposed method maintains strong performance across a range of challenging acquisition conditions, exhibiting robustness to typical artifacts frequently encountered in clinical CTA imaging.

## 4. Discussion and Conclusions

This study presents a novel one-stage multitask network for simultaneous vessel segmentation and centerline extraction, eliminating the need for any pre- and post-processing steps. The proposed method achieved superior accuracy on non-bifurcating vessels compared to both classical and deep learning-based commonly used methods with publicly available implementations. The combination of convolution and graph layers in the decoders enabled direct centerline generation with subvoxel precision, distinguishing our approach from previously developed techniques. Furthermore, we demonstrated the advantages of the multitask learning strategy in terms of both performance and robustness. Our neural network was significantly more robust to image transformations than the classical VMTK method, highlighting its strong potential for fully automated and reliable centerline path tracking.

Previously proposed methods [34,48,49,54] for segmentation and centerline extraction, including multitask neural networks, predominantly rely on intermediate representations or hybrid pipelines rather than end-to-end solutions. Tetteh et al. [34] reduced the centerline detection task to segmentation problem of the input image pixels. In contrast, Yaushev et al. [54] formulated centerline detection as an implicit task. One head of their network generates vessel segmentation masks, while the second head predicts displacement fields from any pixel of the input image to the closest centerline point. Using these predicted displacement fields and segmentation masks, the authors first generate candidate centerline points and then apply a non-maximum suppression technique to refine the point cloud. Although the authors claim that their approach outperforms segmentation-based methods in aortic centerline reconstruction, it requires extensive post-processing steps to obtain final centerline points. Furthermore, none of the aforementioned methods directly generate continuous centerlines. Instead, they produce discrete centerline points that necessitate subsequent connectivity determination. Thus, the proposed method not only effectively overcomes the identified issue but also demonstrates superior performance compared to existing approaches.

The limitations of our method include its reliance on constructing the centerline for a vascular network with a predefined topology, which reduces its flexibility in cases where the topology of the target vessel exhibits significant variability. However, this issue could potentially be addressed by modifying the method, for instance, by abandoning the assumption of predefined connectivity between centerline segments and instead generating only its points. The graph layers utilized in our network, which are based on polyline edges, can be substituted with graph layers that dynamically define connectivity between points. Several k-nearest neighbor-based approaches have been proposed in the literature, for example, to analyze point clouds [72] and particle detector data [73]. Connectivity between these points could then be established using a minimal spanning tree algorithm [74]. Moreover, this might involve employing a differentiable variant of the minimal spanning tree algorithm [75] alongside regularization terms in the loss that penalize discrepancies between the generated and the ground truth centerline topologies. Unlike existing methods that also extract unconnected centerline points, the described modification of our method explicitly incorporates a connectivity method during training and proposes regularization to ensure topological consistency.

Nevertheless, despite the aforementioned limitations, the proposed method can be effectively applied in tasks where the centerline topology is known a priori. For example, in preoperative path planning [76], where the target trajectory is assumed to be unbranched, or in spinal column centerline extraction [77], where the topology is fixed and exhibits minimal variability, our approach is guaranteed to reconstruct the correct structure without requiring post-processing to remove spurious branches or loops. Given that the method demonstrated high accuracy in vessel segmentation and centerline extraction tasks, as well as robustness to input image perturbations, it holds potential for integration into automated frameworks for vascular morphological analysis in clinical practice.

## Figures and Tables

**Table 1:** A comparison of prediction accuracy across different methods.

Task Name		Segmentation	Centerline Extraction			
		VD (%) ↑	SD-1 (%) ↑	SD-3 (%) ↑	HD (mm) ↓	ASSD (mm) ↓
Mass centroid	CM1	-	64.89 ± 11.18	89.17 ± 6.02	8.68 ± 3.72	8.68 ± 3.72
Thinning	CM2	-	58.63 ± 11.28	92.72 ± 8.86	4.88 ± 4.56	1.43 ± 0.68
Kimimaro	CM3	-	66.93 ± 11.92	95.77 ± 2.80	3.67 ± 1.69	1.17 ± 0.30
VMTK *	CM4	-	66.29 ± 10.73	95.99 ± 3.00	3.28 ± 1.62	1.09 ± 0.27
Tetteh et al. [34]	NM1	-	58.70 ± 7.02	95.24 ± 3.59	7.87 ± 17.09	1.51 ± 1.15
Yaushev et al. [54]	NM2	-	56.00 ± 20.00	97.00 ± 4.00	15.00 ± 16.00	1.4 ± 1.1
Proposed NN		91.09 ± 0.02	72.52 ± 8.96	97.65 ± 2.07	2.74 ± 0.81	0.93 ± 0.21
Proposed NN only centerline extraction		-	70.01 ± 10.37	96.71 ± 2.30	5.85 ± 9.12	1.15 ± 0.52
Proposed NN only mask segmentation		90.83 ± 0.02	-	-	-	-

* denotes that metrics are reported exclusively for cases successfully processed by VMTK method.

**Table 2:** A comparison of prediction accuracy under CT image artifacts.

Task Name	Segmentation	Centerline Extraction			
	VD (%) ↑	SD-1 (%) ↑	SD-3 (%) ↑	HD (mm) ↓	ASSD (mm) ↓
Noised data	91.09±0.02	72.37±8.21	97.61±1.89	2.74±0.94	0.93±0.23
Data with calibration artefacts	91.09±0.03	72.69±7.58	97.67±1.75	2.72±0.89	0.92±0.21
Motion blurred data	91.09±0.03	67.56±11.31	95.88±4.78	3.4±2.33	1.08±0.37
Original data	91.09 ± 0.02	72.52 ± 8.96	97.65 ± 2.07	2.74 ± 0.81	0.93 ± 0.21

**Figure A1:**
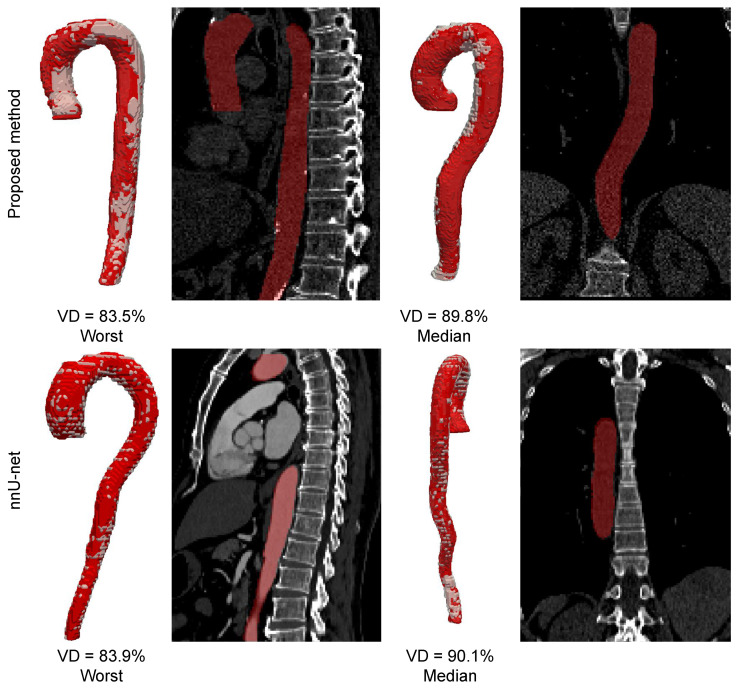
Examples of ground truth segmentation masks (shown in white) and segmentation results (shown in red) for the proposed method and nnU-net.

## Data Availability

The datasets are available in their original repository. For AMOS: https://amos22.grand-challenge.org, accessed on 6 May 2025, LIDC-IDRI: https://www.cancerimagingarchive.net/collection/lidc-idri, accessed on 6 May 2025, and additional annotations: https://github.com/neuro-ml/curve-detection, accessed on 6 May 2025. The source code of the proposed method is available in https://github.com/rostepifanov/paper-vessel-centerline, accessed on 6 June 2025.

## Appendix A

In this section, we investigate how the performance of our network is affected when the selected EfficientNetV2 b0 encoder is replaced with other commonly used convolutional architectures, such as ResNet and DenseNet. All models were trained using the maximum batch size that could fit into GPU memory, without modifying any other training hyperparameters. The results are presented in Table A1. We observe that segmentation accuracy is relatively consistent across different encoder architectures. However, the performance on the centerline reconstruction task varies significantly between encoders. Although DenseNet-121 was the most memory-intensive encoder, it demonstrated inferior performance compared to ResNet-50, despite being trained with the same batch size. On the SD-3 metric, DenseNet-121 achieved a score of 49.25%, substantially lower than the 66.39% attained by ResNet-50. Smaller ResNet variants allowed for larger batch sizes during training. Specifically, ResNet-18 and ResNet-34 supported maximum batch sizes of four and two, respectively. However, ResNet-34 showed slightly lower SD-3 performance than ResNet-50. However, on absolute metrics such as HD and ASSD, ResNet-34 performed noticeably better, indicating that larger batch sizes reduce the variance of centerline point localization errors. While the performance of ResNet-18 was generally comparable to that of EfficientNetV2 b0, the latter consistently achieved slightly better results across all evaluated metrics.

**Table A1:** A comparison of prediction accuracy across different encoder architectures.

Task Name		Segmentation	Centerline Extraction			
Architecture	Max BS ↑	VD (%) ↑	SD-1 (%) ↑	SD-3 (%) ↑	HD (mm) ↓	ASSD (mm) ↓
Resnet 18	4	89.36±2.01	69.74±8.67	97.48±2.41	3.01±1.88	1.01±0.24
Resnet 34	2	87.78±4.56	25.81±13.49	63.98±19.01	8.07±3.44	3.13±1.31
Resnet 50	1	86.41±5.18	29.78±15.89	66.39±25.59	21.27±19.21	4.85±4.22
Densenet 121	1	85.18±6.17	19.01±11.08	49.25±23.76	19.64±11.57	5.51±3.74
EffitientnetV2 b0	4	91.09 ± 0.02	72.52 ± 8.96	97.65 ± 2.07	2.74 ± 0.81	0.93 ± 0.21

## Appendix B

Based on our observations, random initialization does not significantly affect the final performance of the neural network. To quantify this effect, we trained three instances of the network using the same data fold but with different initializations of the centerline points. The results are presented in Table A2, which reports the mean and variance of the averaged performance metrics of the neural networks. The observed variance is relatively low, indicating that the initialization of the centerline points has a minimal impact on the overall network performance.

**Table A2:** The mean and variance of the averaged performance metrics of the neural networks with different polyline initialization in the centerline extraction task.

SD-1 (%) ↑	SD-3 (%) ↑	HD (mm) ↓	ASSD (mm) ↓
72.08±0.38	97.46±0.21	2.69±0.04	0.93±0.04

## Appendix C

According to [54], we employed one-dimensional versions of the following metrics to evaluate the quality of centerline generation: SD −τ, HD, and ASSD. SD −τ was defined as follows:

$$\begin{array}{c} \mathrm{SD}-\tau \left(C_{p},C_{t}\right)=\frac{|\left\{v_{t}\in C_{t}|d\left(v_{t},C_{p}\right)\leq \tau \right\}\left|+\right|\left\{v_{p}\in C_{p}|d\left(v_{p},C_{t}\right)\leq \tau \right\}|}{|C_{t}\left|+\right|C_{p}|}, \end{array}$$

where v∈C denotes iteration over the points of the centerline, d(v,C) is the Euclidean distance between the point *v* and the centerline *C*, and τ is a threshold expressed in millimeters. ASSD was defined as follows:

$$\begin{array}{c} \mathrm{ASSD}\left(C_{p},C_{t}\right)=\frac{1}{|C_{p}|}\sum_{v_{t}\in C_{t}}d\left(v_{t},C_{p}\right)+\frac{1}{|C_{t}|}\sum_{v_{p}\in C_{p}}d\left(v_{p},C_{t}\right). \end{array}$$

HD was defined as follows:

$$\begin{array}{c} \mathrm{HD}\left(C_{p},C_{t}\right)=\max\left\{{\mathrm{perc}}_{v_{t}\in C_{t}}^{95}d\left(v_{t},C_{p}\right),\;{\mathrm{perc}}_{v_{p}\in C_{p}}^{95}d\left(v_{p},C_{t}\right)\right\}, \end{array}$$

where max denotes the maximum value, and ${\mathrm{perc}}^{95}$ refers to the 95th percentile.

## Appendix D

We compared the segmentation quality of the proposed method with the segmentation quality achieved by the nnU-Net method [78]. The nnU-Net is a strong baseline method that automatically configures an optimal convolutional neural network architecture for a given segmentation task based on data analysis. For training, nnU-Net employs the Adam optimizer with an initial learning rate of 3 × $10^{-4}$, and automatically reduces the learning rate every 30 epochs twice if no improvement in performance is observed. One epoch is defined as an iteration over 250 training batches, and training is performed for up to a maximum of 1000 epochs. To augment the training dataset, nnU-Net applies various techniques such as random rotations, random scaling, random elastic deformations, gamma correction augmentation, and mirroring. The results of the nnU-Net method and our proposed method are presented in Table A3. Examples of segmentation results are shown in Figure A1. Based on the experimental results, we conclude that the segmentation performance of both methods is comparable.

**Table A3:** A comparison of prediction accuracy of segmentation across different methods.

	VD (%) ↑
nnU-net	91.93 ± 0.02
Proposed NN	91.09±0.02
Proposed NN only mask segmentation	90.83±0.02
